# Supplementary figures and images for: Caveolin-1 protects endothelial cells from extensive expansion of transcellular tunnel by stiffening the plasma membrane
Source: eLife. 2024 Mar 22;12:RP92078. doi: 10.7554/eLife.92078 (PMC10959525; doi:10.7554/eLife.92078)

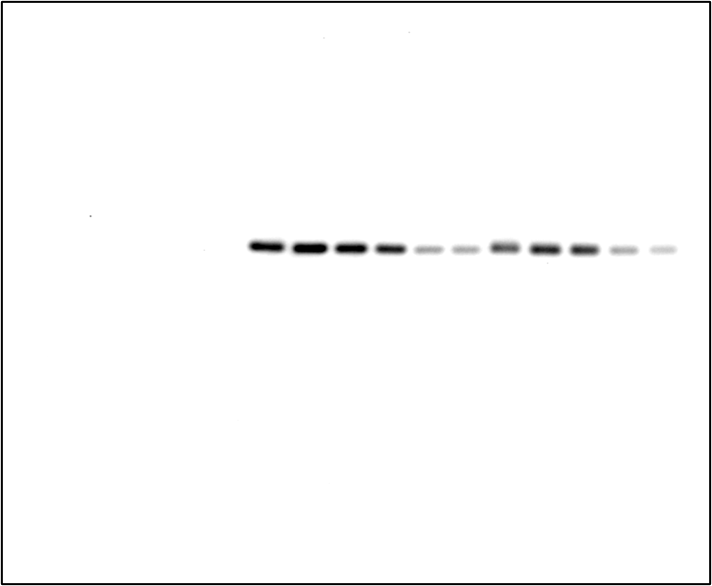

Supplement: Figure 1—figure supplement 1—source data 1. [file elife-92078-fig1-figsupp1-data1.zip › F1-FS1 A CAV1.tif]

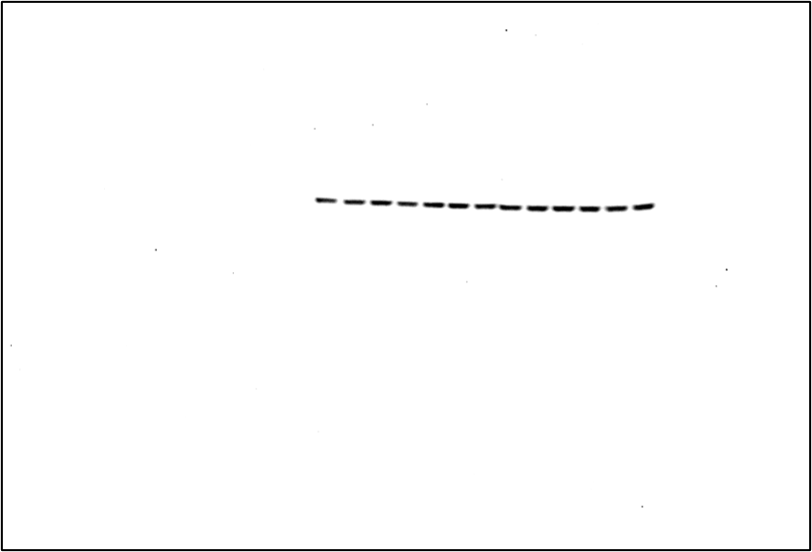

Supplement: Figure 1—figure supplement 1—source data 1. [file elife-92078-fig1-figsupp1-data1.zip › F1-FS1 A GAPDH.tif]

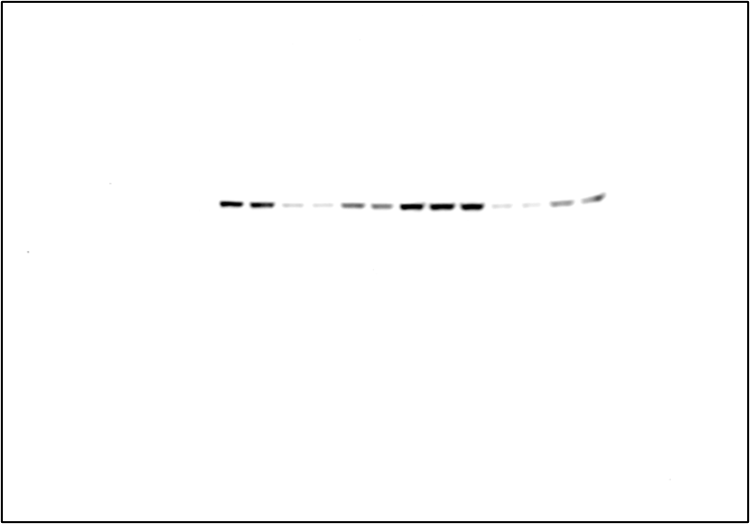

Supplement: Figure 1—figure supplement 1—source data 1. [file elife-92078-fig1-figsupp1-data1.zip › F1-FS1 A PTRF.tif]

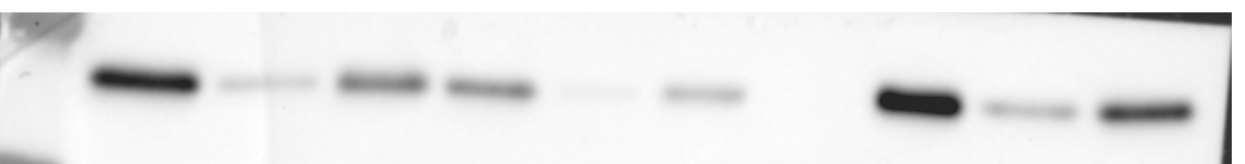

Supplement: Figure 1—figure supplement 1—source data 1. [file elife-92078-fig1-figsupp1-data1.zip › F1-FS1 B CAV1.tif]

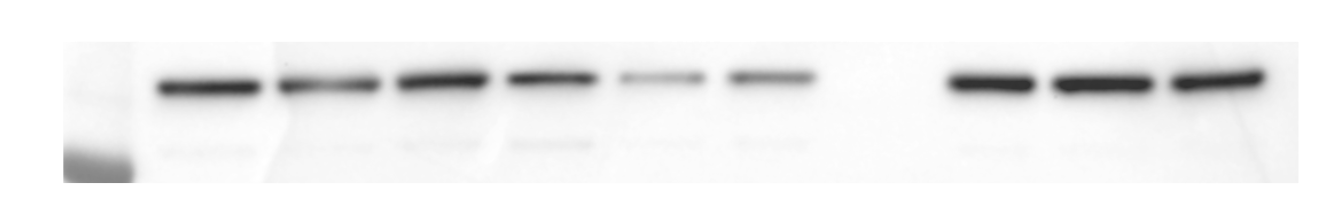

Supplement: Figure 1—figure supplement 1—source data 1. [file elife-92078-fig1-figsupp1-data1.zip › F1-FS1 B GAPDH.tif]

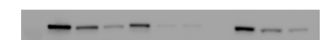

Supplement: Figure 1—figure supplement 1—source data 1. [file elife-92078-fig1-figsupp1-data1.zip › F1-FS1 B PTRF.tif]

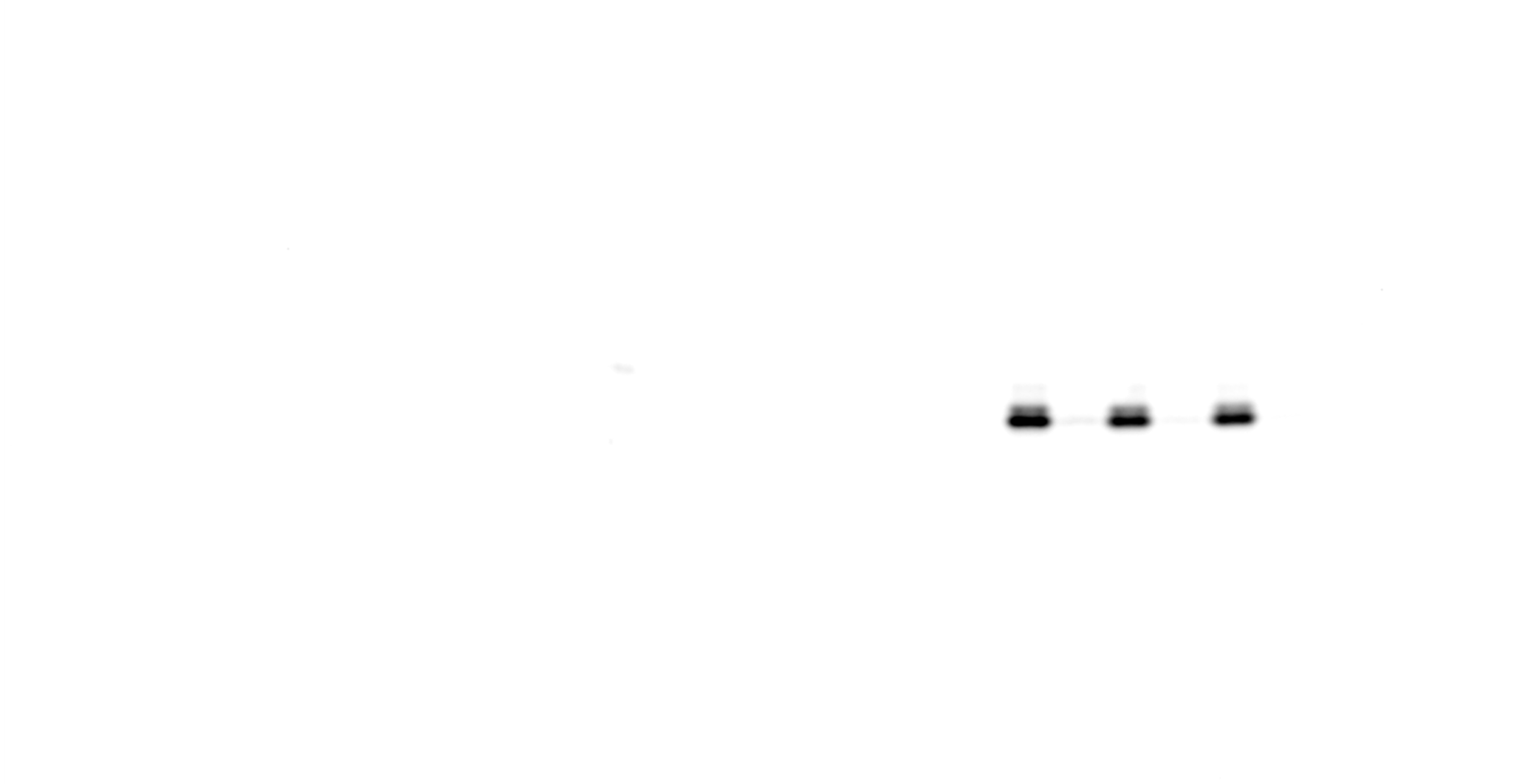

Supplement: Figure 1—figure supplement 1—source data 1. [file elife-92078-fig1-figsupp1-data1.zip › F1-FS1 C biotin ADPr.tif]

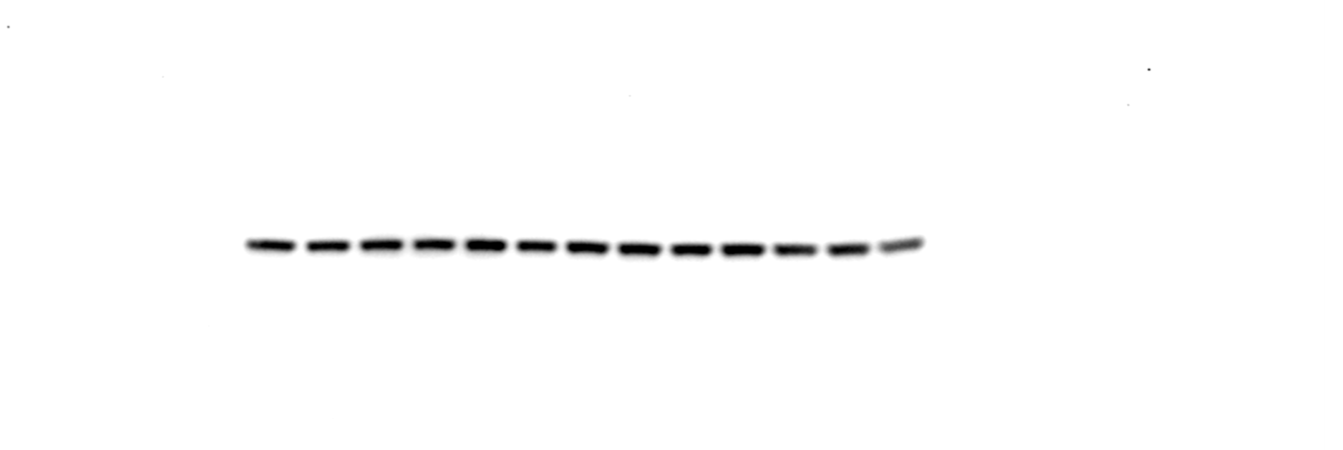

Supplement: Figure 1—figure supplement 1—source data 1. [file elife-92078-fig1-figsupp1-data1.zip › F1-FS1 C GAPDH.tif]

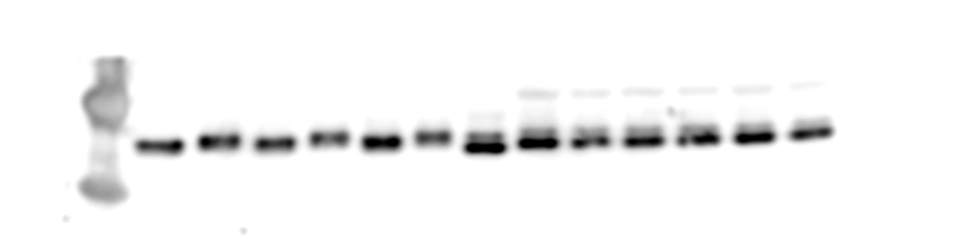

Supplement: Figure 1—figure supplement 1—source data 1. [file elife-92078-fig1-figsupp1-data1.zip › F1-FS1 C RhoA.tif]

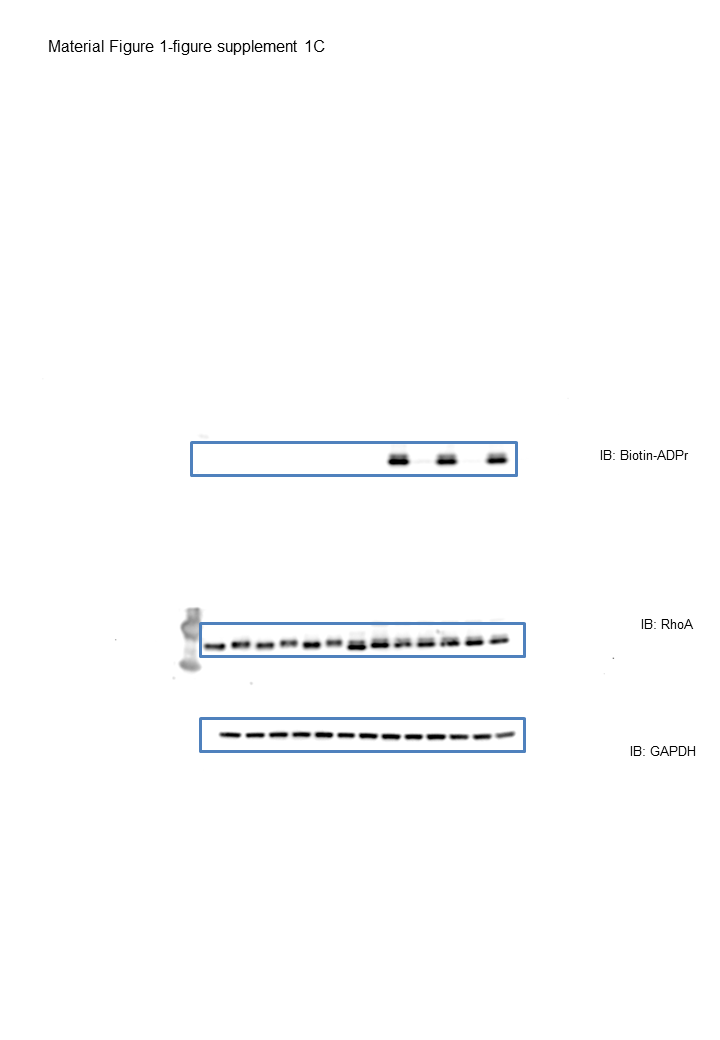

Supplement: Figure 1—figure supplement 1—source data 1. [file elife-92078-fig1-figsupp1-data1.zip › F1-S1C.TIF]

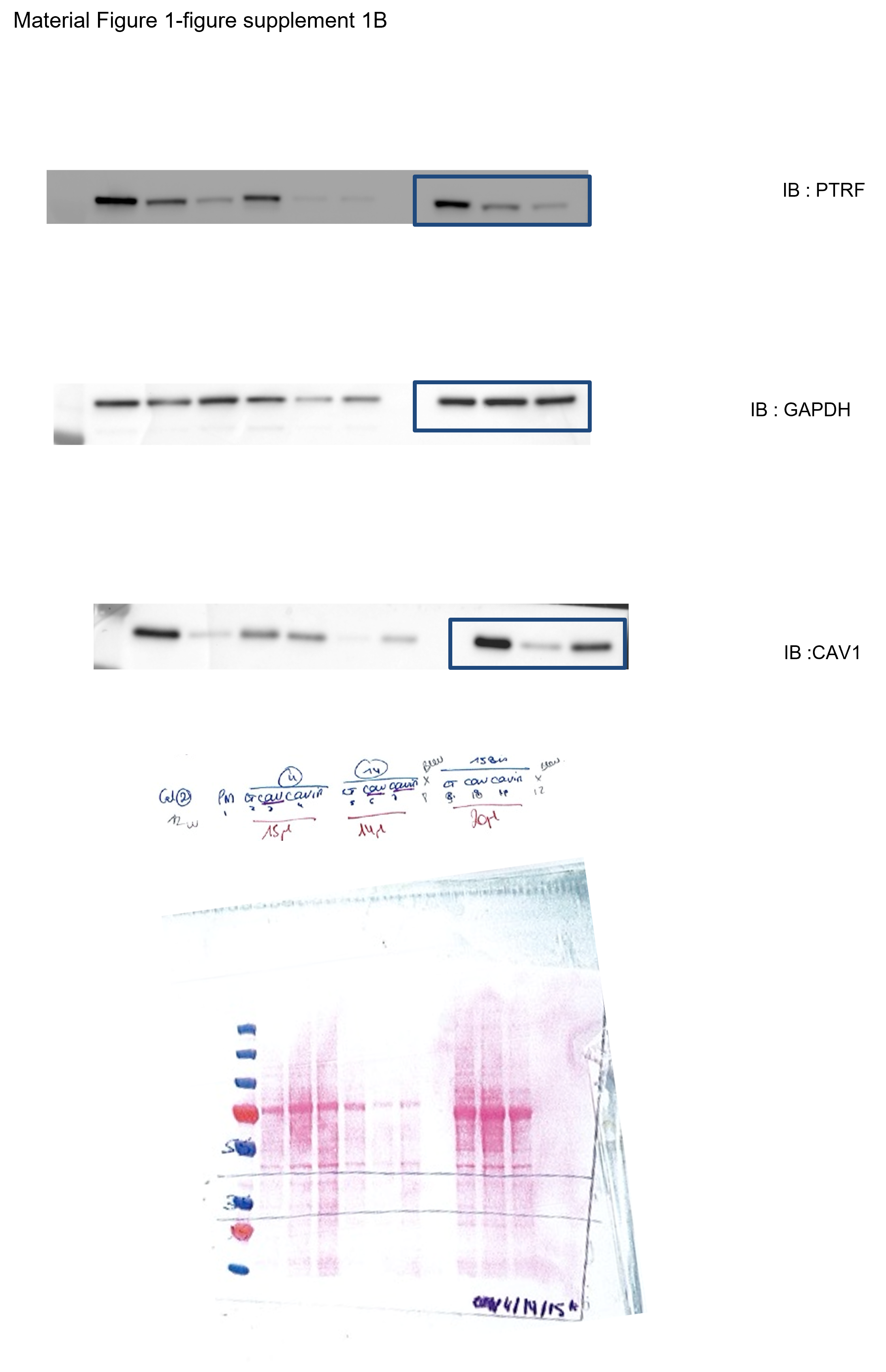

Supplement: Figure 1—figure supplement 1—source data 1. [file elife-92078-fig1-figsupp1-data1.zip › F1-S1B.TIF]

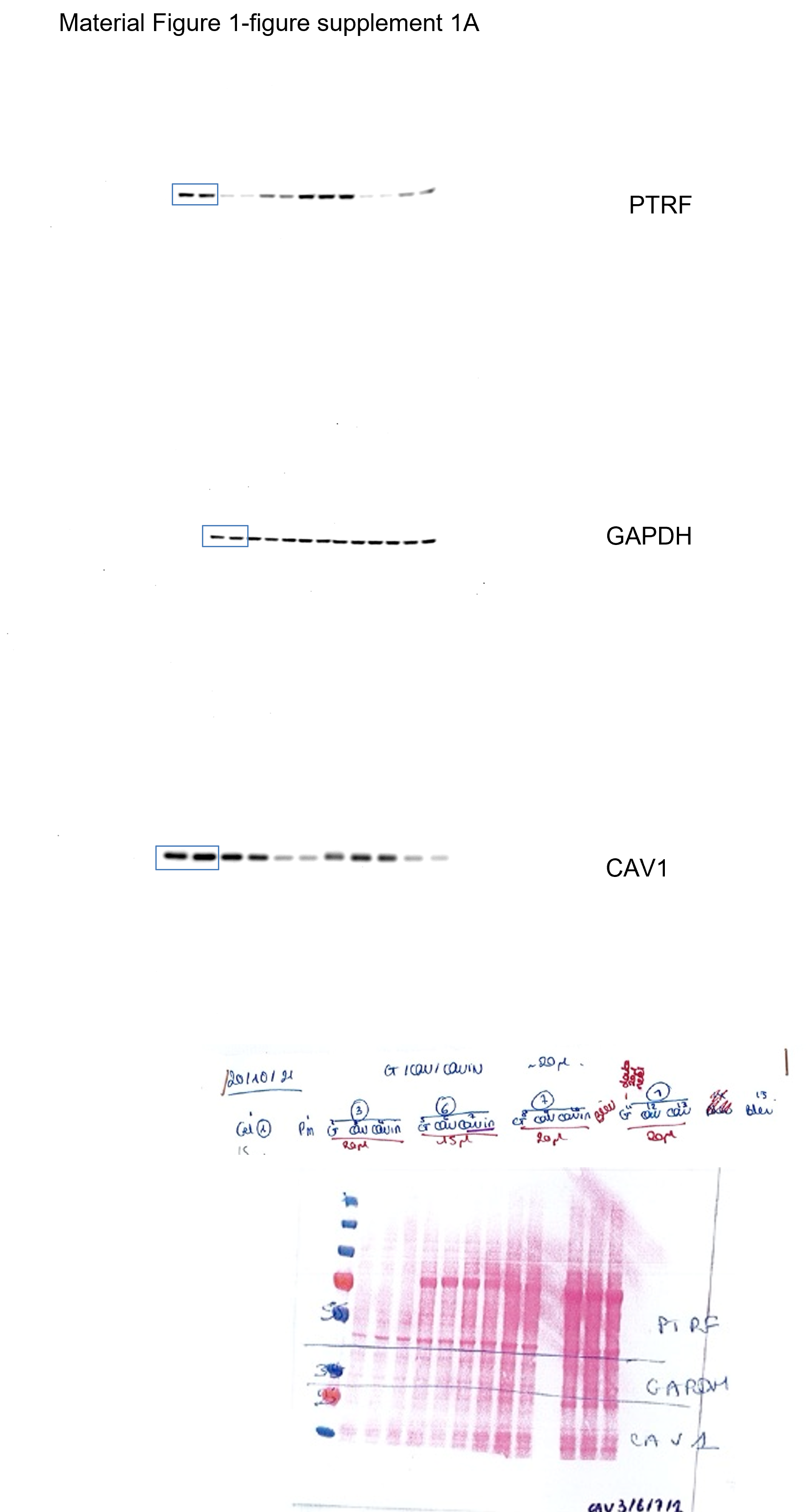

Supplement: Figure 1—figure supplement 1—source data 1. [file elife-92078-fig1-figsupp1-data1.zip › F1-S1A.TIF]

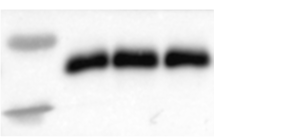

Supplement: Figure 1—figure supplement 3—source data 1. [file elife-92078-fig1-figsupp3-data1.zip › F1-FS3 A CAV1.tif]

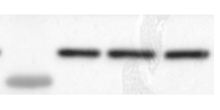

Supplement: Figure 1—figure supplement 3—source data 1. [file elife-92078-fig1-figsupp3-data1.zip › F1-FS3 A GAPDH.tif]

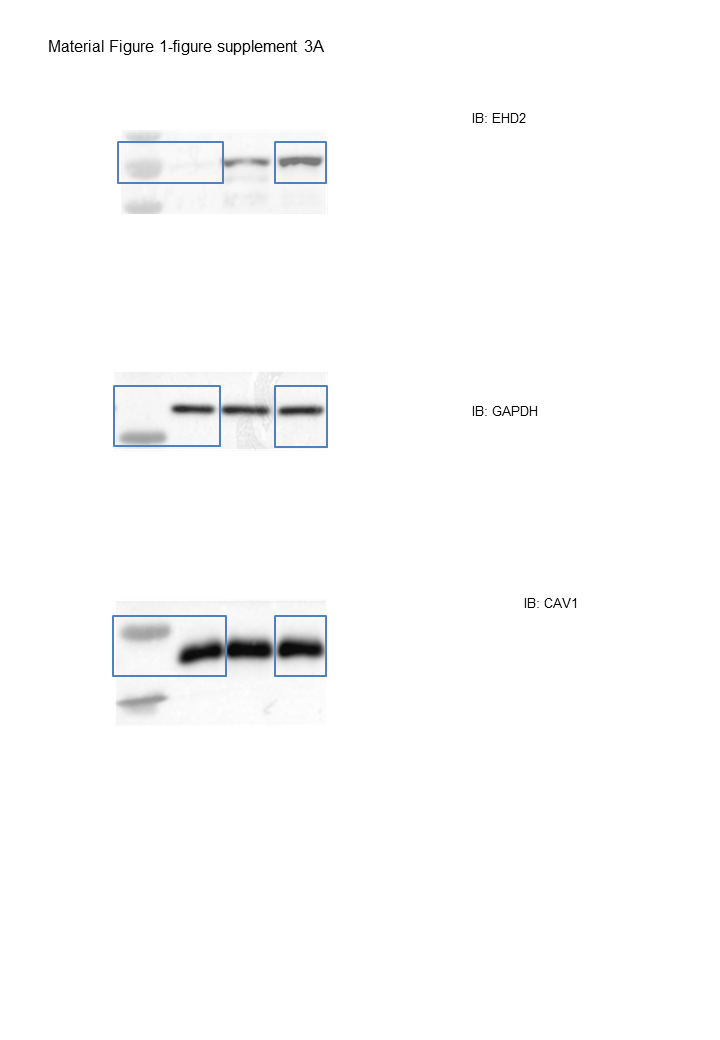

Supplement: Figure 1—figure supplement 3—source data 1. [file elife-92078-fig1-figsupp3-data1.zip › F1-S3A.TIF]

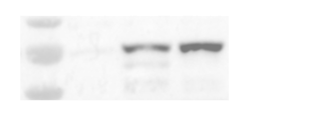

Supplement: Figure 1—figure supplement 3—source data 1. [file elife-92078-fig1-figsupp3-data1.zip › F1-SF3 A EDH2.tif]
